# Translation method is validity evidence for construct equivalence: analysis of secondary data routinely collected during translations of the Health Literacy Questionnaire (HLQ)

**DOI:** 10.1186/s12874-020-00962-8

**Published:** 2020-05-26

**Authors:** Melanie Hawkins, Christina Cheng, Gerald R. Elsworth, Richard H. Osborne

**Affiliations:** 1 School of Health and Social Development, Faculty of Health, Deakin University, Geelong, Australia; 2 Centre for Global Health and Equity, Faculty of Health, Arts and Design, Swinburne University, Postal address: AMDC building, Level 9, Room 907, 453/469-477 Burwood Road, Hawthorn, Australia

**Keywords:** Patient-reported outcomes measure, Construct equivalence, Translation method, Validity testing theory, Validity evidence, Health Literacy Questionnaire, Health literacy

## Abstract

**Background:**

Cross-cultural research with patient-reported outcomes measures (PROMs) assumes that the PROM in the target language will measure the same construct in the same way as the PROM in the source language. Yet translation methods are rarely used to qualitatively maximise construct equivalence or to describe the intents of each item to support common understanding within translation teams. This study aimed to systematically investigate the utility of the Translation Integrity Procedure (TIP), in particular the use of item intent descriptions, to maximise construct equivalence during the translation process, and to demonstrate how documented data from the TIP contributes evidence to a validity argument for construct equivalence between translated and source language PROMs.

**Methods:**

Analysis of secondary data was conducted on routinely collected data in TIP Management Grids of translations (*n* = 9) of the Health Literacy Questionnaire (HLQ) that took place between August 2014 and August 2015: Arabic, Czech, French (Canada), French (France), Hindi, Indonesian, Slovak, Somali and Spanish (Argentina). Two researchers initially independently deductively coded the data to nine common types of translation errors. Round two of coding included an identified 10th code. Coded data were compared for discrepancies, and checked when needed with a third researcher for final code allocation.

**Results:**

Across the nine translations, 259 changes were made to provisional forward translations and were coded into 10 types of errors. Most frequently coded errors were Complex word or phrase (*n* = 99), Semantic (*n* = 54) and Grammar (*n* = 27). Errors coded least frequently were Cultural errors (*n* = 7) and Printed errors (*n* = 5).

**Conclusions:**

To advance PROM validation practice, this study investigated a documented translation method that includes the careful specification of descriptions of item intents. Assumptions that translated PROMs have construct equivalence between linguistic contexts can be incorrect due to errors in translation. Of particular concern was the use of high level complex words by translators, which, if undetected, could cause flawed interpretation of data from people with low literacy. Item intent descriptions can support translations to maximise construct equivalence, and documented translation data can contribute evidence to justify score interpretation and use of translated PROMS in new linguistic contexts.

## Background

Cross-cultural research often requires the translation of a patient-reported outcome measure^1^ (PROM) from one linguistic context to another. Core to the process of validation is the assumption that the PROM in the target language will measure the same construct in the same way as the PROM in the source language [1–10]. In cross-cultural research, this essential requirement is typically referred to as construct equivalence [8, 11], which is defined in this paper as ‘the degree to which a construct measured by a test in one cultural or linguistic group is comparable to the construct measured by the same test in a different cultural or linguistic group’ [10]. Confirmation of construct equivalence is usually judged post translation by the statistical criteria of measurement equivalence or invariance [12–17]. However, threats to construct equivalence need to be minimised during the translation process [10, 18, 19]. Yet translation methods seldom tackle construct equivalence [20] and there is limited recommendation for the use of qualitative research methods to investigate ways to maximise construct equivalence during translation [3, 7]. Furthermore, few translation guidelines suggest the use of item intents, as described by the PROM developer, to enable translation team members to have a common understanding of item meanings [19, 21]. Along with a multi-step translation and central review process, detailed descriptions of item intents can support translation teams to maximise construct equivalence while maintaining the linguistic and cultural veracity of the target language. Along with post-translation qualitative and quantitative evidence, systematic documentation and qualitative analysis of reasons for translation decisions in the pursuit of construct equivalence contribute evidence for an argument about the validity of score interpretation and use in the new linguistic context [3, 10, 18, 22–24]. Qualitative data provide an important source of possible explanations for why statistical evidence for non-invariance of items might be occurring, and may also point the way to remedying the problem.

### Approaching construct equivalence

Construct equivalence begins with item equivalence [25, 26]. PROM constructs are represented by scales, which consist of several items, each of which should be carefully developed and selected to capture a specific element of the construct [27]. The goal of a translation is to ensure as closely as possible that, collectively, all the translated items in a scale will measure the construct in the same way as the source language items measure the construct [28–30]. Herdman et al. [11, 31] defined three approaches to cross-cultural construct equivalence:

1. An absolutist approach (i.e., that culture has minimal effect on the construct being measured)
2. A universalist approach (i.e., that culture will have some effect on the construct being measured)
3. A relativist approach (i.e., that culture will have substantial effect on the construct being measured and so standard tools cannot be used across languages and cultures)

While Herdman et al. present these three approaches as discrete categories, we have found it helpful to view them as lying along a continuum. We consider translation of construct-based PROMs to draw largely on the universalist approach while acknowledging that most translators will include some absolutist assumptions about the extent to which the constructs embodied in the source PROM will be relevant in the target culture. Thus, we advocate that although cultural variation needs to be accounted for, every effort should be made during the translation process to maximise construct equivalence between the target and source language PROMs. For example, item intent descriptions explain the meanings of source language items and provide guidance for translators about the best choice of words and phrases in the target language. Of course, assessment of the applicability of a construct in a target language and culture should always be considered prior to commencing translation [19, 20, 25, 31–33].

### Threats to construct validity

Messick suggested two important threats to construct validity: construct underrepresentation and construct irrelevant variance [24]. Each introduces different sources of bias that may systematically raise or lower the scores of the intended respondents and result in inappropriate interpretation and use of scores [9]. Construct underrepresentation can be introduced if there are important facets of the construct that are present in the new culture that were not present in the source culture (e.g., aspects of support and information from family and community in a communal culture that are not present in a more individualistic culture where the PROM was developed). Construct irrelevant variance can be introduced if there are common individual or cultural factors in the new language context that are associated with, for example, the way people respond to questionnaires generally. One or both of these sources of bias can affect the extent to which inferences drawn from the data of a translated PROM are valid for the intended purpose [6, 33].

### Validity testing theory

The authoritative reference for contemporary validity testing theory is the *Standards for Psychological and Educational Testing* (referred to hereon as the *Standards*) [10]. The *Standards* provides a clear theoretical foundation for validation practice [34] and outlines validation criteria for developers and users of measurement instruments who interpret, evaluate, and use the results of measurement with those instruments [10, 18]. Successive publications about validity testing theory have long held that validation is a *process* of evaluating validity evidence to determine the quality and credibility of inferences made from test scores [18, 22–24, 35–48]. In other words, validity does not refer to a measurement instrument but to the extent to which evidence and theory support the interpretation of its data for an intended purpose [10, 49].

Despite publication of translation guidelines [1, 19, 21, 30, 50–52], theory to guide translation practice is limited. The *Standards* outlines five sources of validity evidence (Table 1) that provide a theoretical framework that can be applied to the translation of PROMs to guide generation of evidence and development of a validity argument for score interpretation and use. The *Standards* states that translation method contributes to construct equivalence and asserts that data generated during translation contribute validity evidence for interpretation and use of scores from translated tests (Standard 3.12, p.68 and Standard 7.6, p.127) [10]. As such, the validity of decisions using data from a translated PROM must consider evidence for the translation method [32, 53]. A simple statement in a publication that a best practice translation method was used (and citation of appropriate references) does not demonstrate validity evidence for construct equivalence between PROMs. Transparency through publication of process data from a recommended translation method can provide evidence for an argument that a translation method has been rigorously implemented and has contributed to maximising construct equivalence between languages [19, 53].

**Table 1:** Five sources of validity evidence from the Standards of Educational and Psychological Testing (2014)

**1.**	**Evidence based on test content** The relationship of the item themes, wording and format with the intended construct, including administration process.
**2.**	**Evidence based on response processes** The cognitive processes and interpretation of items by respondents and users, as measured against the intended construct.
**3.**	**Evidence based on internal structure** The extent to which item interrelationships conform to the intended construct.
**4.**	**Evidence based on relations to other variables** The pattern of relationships of test scores to external variables as predicted by the intended construct.
**5.**	**Evidence for validity and the consequences of testing** Intended and unintended consequences, as can be traced to a source of invalidity such as construct underrepresentation or construct-irrelevant variance.

### Aim of this study

The Translation Integrity Procedure (TIP) is a translation method that we developed to qualitatively pursue construct equivalence between translated and source language PROMs by using item intent descriptions as the foundation of the translation (Additional file 1). The TIP has evolved over years of practice as we strove to get conceptually equivalent items and constructs across different PROMs and in many languages [54–56]. For each translation, the process from first forward to final consensus translation is guided by the item intents, and this process is documented in the TIP Management Grid. The aim of this study was twofold:

1. To systematically investigate the utility of the TIP, in particular the use of item intent descriptions, to maximise construct equivalence during the translation process.
2. To demonstrate that qualitative analysis and publication of documented data from a translation process contributes evidence to a validity argument for construct equivalence between translated and source language PROMs.

## Methods

### The study design

**Fig. 1:**
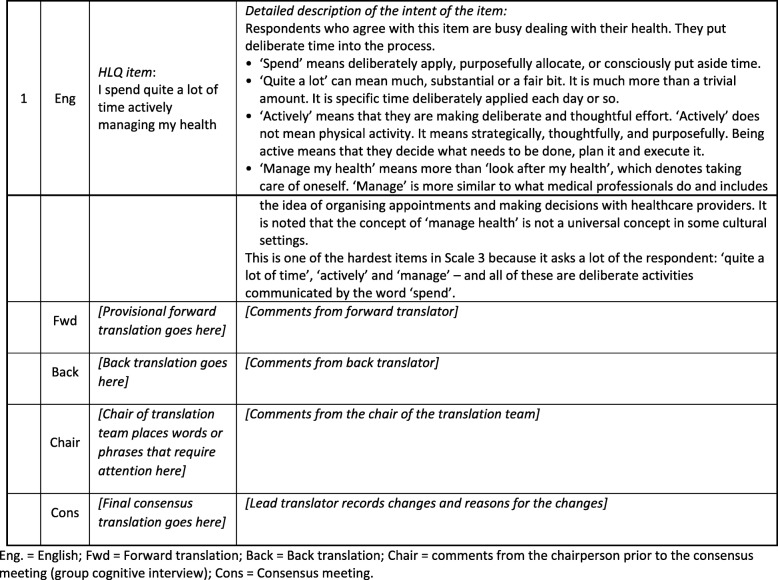
Example of the format of the Translation Integrity Procedure (TIP) Management Grid Eng. = English; Fwd = Forward translation; Back = Back translation; Chair = comments from the chairperson prior to the consensus meeting (group cognitive interview); Cons = Consensus meeting

This study was a secondary data analysis of a convenience sample of routinely collected data in TIP Management Grids of translations (*n* = 9) of the Health Literacy Questionnaire (HLQ) [57] that took place during the study period of August 2014 to August 2015: Arabic, Czech, French (Canada), French (France), Hindi, Indonesian, Slovak, Somali and Spanish (Argentina). The TIP Management Grid is the focal document for HLQ translations and contains the HLQ items, the descriptions of the item intents, and the forward, back and final translations. See Fig. 1 for an example of the format of the TIP Management Grid including an example HLQ item and item intent description.

### The translation process

The translation team consists of the forward translator and one other translator (either the second forward translator or the back translator), bilingual health workers and/or researchers, and patients or local consumers/consumer advocates who are native speakers of the target language. Formal qualifications for translators are expected but not required for every translator for each target language. More important is the capacity of a translator to value the use of the item intents, to have command of their native tongue including vernacular and cultural understanding, and be able to engage with the chairperson in the consensus meeting. The back translator, as a native speaker of English and fluent speaker of the target language, is an important support to the chairperson to help negotiate the nuances of meanings in the English items with the forward translator [25]. Translators are informed that target respondents to the PROM are potentially people with low literacy and low exposure to health and health care so words and phrases in the translation must be those used in everyday language. The chairperson leads the translation team in the group cognitive interview and is usually an author of the PROM being translated but can be another person who is deeply familiar with the items and purpose of the PROM, and experienced in and dedicated to optimising construct equivalence between source and target language PROMs.

The translation team is briefed about the TIP including adherence to the item intents during the translation process. Two forward translators independently translate the items to the target language and then confer to reach consensus on the provisional forward translation, consulting with the rest of the translation team if required. The back translator is blind to the source language items and item intents. A back translation does not add to the quality of a translation [3, 30, 58, 59]. However, it is useful for a PROM developer or translation lead who does not speak the target language (but who is deeply familiar with the items and purpose of the questionnaire) and who needs to confer with the translation team about the translated items. The provisional forward translation and the back translation are entered into the Management Grid along with commentary from translators, and sent to the chairperson of the group cognitive interview. The chairperson reviews the back translation and records comments or questions in the Management Grid about potentially problematic words or phrases. The Management Grid with the chairperson’s comments is returned to the translators for discussion in relation to the item intents in the group cognitive interview. The lead forward translator records all changes to the provisional forward translation, and the reasons for the changes, in the Management Grid. Additional file 1 contains the TIP document. It needs to be noted that as a result of this study, the TIP recommendation for two forward translators to do independent translations and then confer was changed to one independent forward translation with the second translator checking the forward translation against the item intents, then both translators conferring about differences. Additional file 1 contains this update to the TIP method but in this study there were two translators who did independent forward translations.

The translation consensus meeting is conducted like a group cognitive interview in much the same way as Sidani et al. used group cognitive interviews for forward translation (p.140) [20]. During the group cognitive interview, close attention is paid to the item intents to locate words, phrases or concepts in the forward translation that are incorrect or require changes to achieve the most accurate, and linguistically- and culturally-appropriate translation possible. The lead translator records all changes made to the forward translation and the reasons for the changes in the Management Grid. The chairperson for the 9 group cognitive interviews in this study was an author of the HLQ and of this paper (RHO), and each interview took between 3 and 4 h.

### The Health Literacy Questionnaire (HLQ)

The HLQ was designed using a grounded, validity-driven approach [27] and was initially tested in diverse samples of individuals recruited from urban and regional health services in Victoria, Australia. The HLQ was found to have strong construct validity, reliability, and acceptability to clients and clinicians in this context [57]. The purpose of the HLQ is to measure the multi-dimensional concept of health literacy [60]. The HLQ consists of 44 items within 9 scales, each scale comprising 4 to 6 items. The scales have high and low descriptors to define the scope of the element of health literacy that the scale represents (Table 2), and every item has a description of its intent to define its purpose and place within the scale [57]. The HLQ item intents explain the intended meaning of each item and provide translators with information about the conceptual basis of the items and explanations of, or synonyms for, words and phrases within each item. Translators are asked to not only seek excellence in the technical translation and cultural adaptation of items but also to strive for equivalent meaning and difficulty [61, 62]. Each scale score is interpreted within the bounds of the high and low scale descriptors as an independent element of the health literacy construct. The intended interpretation of the 9 HLQ scale scores is to evaluate a profile of the health literacy strengths and challenges of groups and individuals, and to indicate where health organisation or client/service health literacy interventions may be needed [57, 63–65]. The HLQ has been translated into more than 30 languages and is licenced to many organisations around the world. Validity evidence is accumulating to support interpretations of HLQ scores for individual clients [66], for diverse populations [54–56, 65, 67–71], and for population health surveys [72–76]. However, further evidence is required to support the validity of interpretations of HLQ scores for decision making in different population, cultural and linguistic contexts.

**Table 2:** Health Literacy Questionnaire scales and high and low descriptors

Scale number and name	Interpretation – what do the scale scores mean?
**1. Feeling understood and supported by healthcare providers**	**High**: Has an established relationship with at least one healthcare provider who knows them well and who they trust to provide useful advice and information and to assist them to understand information and make decisions about their health. **Low**: People who are low on this domain are unable to engage with doctors and other healthcare providers. They don’t have a regular healthcare provider and/or have difficulty trusting healthcare providers as a source of information and/or advice.
**2. Having sufficient information to manage my health**	**High**: Feels confident that they have all the information that they need to live with and manage their condition and to make decisions. **Low**: Feels that there are many gaps in their knowledge and that they don’t have the information they need to live with and manage their health concerns.
**3. Actively managing my health**	**High**: Recognise the importance of and are able to take responsibility for their own health. They proactively engage in their own care and make their own decisions about their health. **Low**: People with low levels don’t see their health as their responsibility, they are not engaged in their healthcare and regard healthcare as something that is done to them.
**4. Social support for health**	**High**: A person’s social system provides them with all the support they want or need. **Low**: Completely alone and unsupported.
**5. Appraisal of health information**	**High**: Able to identify good information and reliable sources of information. They can resolve conflicting information by themselves or with help from others. **Low**: No matter how hard they try, they cannot understand most health information and get confused when there is conflicting information.
**6. Ability to actively engage with healthcare providers**	**High**: Is proactive about their health and feels in control in relationships with healthcare providers. Is able to seek advice from additional health care providers when necessary. They keep going until they get what they want. Empowered. **Low**: Is passive in their approach to health care, inactive, i.e., they do not proactively seek or clarify information and advice and/or service options. They accept information without question. Unable to ask questions to get information or to clarify what they don’t understand. They accept what is offered without seeking to ensure that it meets their needs. Feel unable to share concerns.
**7. Navigating the healthcare system**	**High**: Able to find out about services and supports so they get all their needs met. Able to advocate on their own behalf at the system and service level. **Low**: Unable to advocate on their own behalf and unable to find someone who can help them use the healthcare system to address their health needs. Do not look beyond obvious resources and have a limited understanding of what is available and what they are entitled to.
**8. Ability to find good health information**	**High**: Is an ‘information explorer’. Actively uses a diverse range of sources to find information and is up to date. **Low**: Cannot access health information when required. Is dependent on others to offer information.
**9. Understand health information well enough to know what to do**	**High**: Is able to understand all written information (including numerical information) in relation to their health and able to write appropriately on forms where required. **Low**: Has problems understanding any written health information or instructions about treatments or medications. Unable to read or write well enough to complete medical forms.

### Data analysis

Coding was conducted after translations were finalised when the translators provided the Management Grids in which they had written documentation of the changes made during the group cognitive interviews and the reasons for the changes. The focus of the coding was the reasons why changes were made because these defined the cause of the translation errors detected during the group interview.

Prior to coding, a list of common types of translation errors was made [77] and used as a preliminary coding framework. Nine common error types were identified: Cultural, Grammar, Idiom/literal meaning, Measurement, Printed errors, Sematic, Unit of meaning – additional, Unit of meaning – omission, Unit of meaning – substitution. These were assigned definitions to support systematic and consistent coding. Coders independently identified a 10th code: Complex word or phrase. See Table 3.

**Table 3:** Coding framework and definitions

Codes	Definitions
**1. Complex word or phrase**	Translated word or phrase is changed to replace an inappropriate, technical, complex or difficult to understand word or phrase to improve flow or to make the sentence more easily understood.
**2. Cultural**	Translated word or phrase is adapted to be more culturally appropriate while maintaining semantic and measurement equivalence with English items.
**3. Grammar**	Instances when incorrect grammar is detected in the forward translation. For example, incorrect verb tenses or verb forms, or incorrect declension of nouns, pronouns, or adjectives.
**4. Idiom/literal meaning**	Instances when the English item contains a word or an expression that has a literal meaning that is different from the meaning it intends to convey. e.g., Part 1, Item 1. ‘I feel I have good information about health’, where ‘I feel’ is better translated as ‘I believe’ or ‘I think’.
**5. Measurement**	Translated word or phrase is altered to better match the strength of the English expression or the measurement distance between English items, while maintaining semantic equivalence with English words and phrases, and cultural appropriateness in the target language.
**6. Printed errors (e.g., spelling, punctuation, typographical errors)**	Instances when punctuation or typographical errors (including spelling errors) are detected in the forward translation.
**7. Semantic**	Translated word or phrase is altered to better match the English meaning while maintaining measurement equivalence with English items and cultural appropriateness in the target language.
**8. Unit of meaning – Additional**	Instances when the translator adds meaning to the translation that was not in the original English.
**9. Unit of meaning – Omission**	Instances when the translator omits meaning from the translation that was in the original English.
**10. Unit of meaning – Substitution**	Instances when the translator uses a word or phrase in the translation that is a different meaning from the original English.

**Fig. 2:**
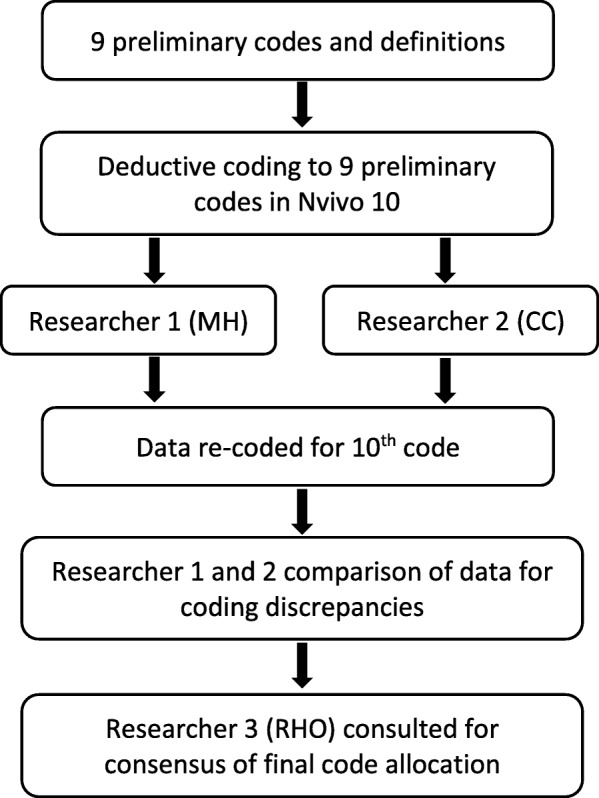
Data analysis method

Two researchers (MH and CC) initially independently coded the data to the nine preliminary codes, and then conducted a second round of coding to include the 10th code. Researchers 1 and 2 compared coded data for discrepancies. Where coding consensus was not achieved, a third researcher (RHO) was consulted for final code allocation (26/259 codes or 10% of coded data) (Fig. 2).

### Ethics

This project was granted exemption from ethics by the Deakin University Human Research Ethics Committee (DUHREC project: 2015–205).

## Results

During the 9 group cognitive interviews, there were 259 changes made to provisional forward translations. These changes were coded into 10 types of errors (see final rows of Tables 4 and 5 for totals). Agreement between coders was 90% with a third researcher consulted about final code allocation for the remaining 10% (26/259). The types of errors that were coded most frequently were Complex word or phrase (*n* = 99), Semantic (*n* = 54) and Grammar (*n* = 27). The errors that were coded least frequently were Cultural errors (*n* = 7) and Printed errors (*n* = 5).

**Table 4:** Error frequency per language

Language	Complex word or phrase	Cultural	Grammar	Idiom/literal meaning	Measurement	Printed error	Semantic	Unit of meaning - Additional	Unit of meaning - Omission	Unit of meaning - Substitution	Total errors *per language*
**Arabic**	1	0	0	1	1	0	2	0	0	0	**5**
**Czech**	1	1	1	1	0	0	2	2	3	2	**13**
**French (Canada)**	8	0	5	3	0	0	1	1	0	0	**18**
**French (France)**	16	1	2	2	5	0	7	5	1	4	**43**
**Hindi**	34	3	0	1	0	0	6	1	0	2	**47**
**Indonesian**	21	0	1	1	1	0	12	0	0	1	**37**
**Slovak**	3	2	6	3	1	1	6	1	4	5	**32**
**Somali**	0	0	7	0	1	4	7	3	2	2	**26**
**Spanish (Argentina)**	15	0	5	1	2	0	11	0	3	1	**38**
**Total errors per code*across languages***	**99**	**7**	**27**	**13**	**11**	**5**	**54**	**13**	**13**	**17**	**259**

**Table 5:** Error frequency per Health Literacy Questionnaire (HLQ) scale

HLQ scales	Complex word or phrase	Cultural	Grammar	Idiom/literal meaning	Measurement	Printed error	Semantic	Unit of meaning - Additional	Unit of meaning - Omission	Unit of meaning - Substitution	Total errors *per scale*
**1. Feeling understood and supported by healthcare providers**	6	1	3	0	3	0	5	2	1	0	**21**
**2. Having sufficient information to manage my health**	10	0	5	2	1	0	4	0	1	1	**24**
**3. Actively managing my health**	8	1	1	8	5	2	10	2	1	2	**40**
**4. Social support for health**	12	2	3	0	0	1	4	3	1	8	**34**
**5. Appraisal of health information**	17	0	4	0	0	0	2	1	1	1	**26**
**6. Ability to actively engage with healthcare providers**	12	0	3	3	1	1	9	2	4	0	**35**
**7. Navigating the healthcare system**	13	0	3	0	0	0	9	0	1	2	**28**
**8. Ability to find good health information**	13	0	2	0	0	1	9	0	2	1	**28**
**9. Understand health information well enough to know what to do**	8	3	3	0	1	0	2	3	1	2	**23**
**Total errors per code*across scales***	**99**	**7**	**27**	**13**	**11**	**5**	**54**	**13**	**13**	**17**	**259**

### Error frequency for languages

The number of errors detected per language ranged from 5 to 47 (Table 4). The language with the highest number of errors in the forward translation was Hindi (*n* = 47), 34 (72%) of which were coded as Complex word or phrase. Despite the TIP instructions, the lead Hindi translator had used high level language in the forward translation, which required much negotiation and many changes during the group cognitive interview. French (France) also had many changes made to the forward translation (*n* = 43; 16 (37%) Complex word or phrase). There were 9 people from different areas of France who attended the group interview, which resulted in an in-depth discussion about words and phrases that would be suitable across France and across education levels. Spanish (Argentina) required 38 changes (15 or 39% Complex word or phrase) to the forward translation and these were largely informed by input from patients from the target population who attended the group cognitive interview. The Indonesian translation also had a high number of changes to the forward translation (*n* = 37; 21 (57%) Complex word or phrase). This translation followed a different path to the other translations. After generating the provisional forward translation, the Indonesian team tested it with locals and this feedback was incorporated into the Management Grid for the group cognitive interview. The Arabic HLQ had the least number of changes to a forward translation (*n* = 5). Two translators only were present at this group interview, which meant that without a local health researcher, health worker or other local bilingual attendee, the breadth of the discussion was limited.

### Error frequency for Health Literacy Questionnaire (HLQ) scales

The number of errors detected per HLQ scale ranged from 21 to 40 (Table 5). The highest numbers of errors were seen in Scale 3. Actively managing my health (*n* = 40), Scale 6. Ability to actively engage with healthcare providers (*n* = 35), and Scale 4. Social support for health (*n* = 34). Scale 1. Feeling understood and supported by healthcare providers had the lowest number of errors coded (*n* = 21).

Scale 3 had the highest number of Semantic (*n* = 10), Idiom/literal meaning (*n* = 8) and Measurement (*n* = 5) errors of all scales. Semantic errors were detected in every item in Scale 3. There are 2 items of the 5 items in this scale that use the word *things* and adjustment of the meaning in the translations for this concept comprised 4 of the 10 Semantic errors detected. The 8 Idiom/literal meaning errors detected in Scale 3 were all related to the item about setting goals for *health and fitness*. These words go together in English and are explained in the item intent as meaning ‘an optimum fitness level depending on a person’s health problems and health circumstances’. However, this is not the meaning that this phrase has in many other languages. For example, in the Arabic group cognitive interview, the word *fitness* was found to imply being very healthy such that you could run a long distance and not get tired. The Indonesian translators explained that the concept of *fitness* is associated with modern Western living and would not be widely known or understood by all generations in Indonesia. The word *fitness* was left out of all 9 final translations. Translators in all the group interviews agreed that by just using the word *health*, the translations of this item maintained the meaning of setting goals about *health and fitness*. The 5 Measurement errors in Scale 3 were all to do with the item in this scale about spending *quite a lot of time* actively managing health: this was an issue to do with the translation of *quite* to a word with the same strength of expression, or equivalence of difficulty, in other languages.

The main errors coded in Scale 6 were Complex word or phrase (*n* = 12) (words were simplified to be better suited to people with low education) and Semantic (*n* = 9) (translated words of similar meaning were changed to gain a more precise meaning of the English words, as guided by the item intents).

Scale 4 also had a high number of Complex word or phrase errors (n = 12) and some Semantic errors (*n* = 4) but had the highest number of Unit of meaning – Substitution errors (*n* = 8). This was primarily to do with the substitution in forward translations of *and* for *or* in an item that asks about social support from *family or friends*.

### Cross tabulation of results across languages and scales

Complex word or phrase was the most frequently coded error across languages (*n* = 99; range 1–34) and HLQ scales (range 6–17) and always indicated that translators had not used words or phrases that were familiar to or easily understood by people of all education levels. Complex words or phrases were detected most often in Hindi, Indonesian, French (France) and Spanish (Argentina). In 5 scales of the Hindi translation (Scales 2, 5, 6, 7 and 8), every item had to be altered to be easier to read for someone with low education or literacy. The feedback from local people during the field testing of the Indonesian provisional forward translation was aligned in the group cognitive interview with the item intents, and informed changes to the higher level language used by the forward translator. For the French (France) translation (Scale 7), *rencontrer* (encounter or meet) was changed to *voir* (to see) to keep to more commonly-used vocabulary in an item about getting to see healthcare professionals. In the Spanish (Argentina) translation of an item about getting health information (Scale 8), *obtener* (to obtain) was changed to *conseguir* (to obtain or get), which translators explained is a colloquial expression and more accessible to more people.

Semantic errors were also frequently coded across languages (*n* = 54; range 1–12) and HLQ scales (*n* = 54; range 2–10). Indonesian (*n* = 12) and Spanish (Argentina) (*n* = 11) had the highest numbers of errors coded as Semantic. The 12 detected Semantic errors in Indonesian were spread across 5 HLQ scales (Scales 3, 5, 6, 7 and 8). The Indonesian Scale 8 contained 5 of the 12 semantic errors and these were all to do with the concept of finding or getting and obtaining health information. The word *mencari* (to look for) was changed to *menemukan* (to search and discover) because the item intents for this scale describe the English words *find* (used in two items) and *get* (used in 3 items) as having the concept of identifying, locating and obtaining health information, with the rest of the content of each item representing a range of difficulty with this task.

This study also highlighted that the English idiom *I feel* (Scales 2, 4 and 6) can be difficult to translate to other languages (Czech, both French translations, Slovak and Somali). The description of the intended meaning of items containing *I feel* refer to it meaning that a respondent has a sense of or an impression of something. It is not to be translated as *I believe* nor as *I am certain*. It is noted in the intent descriptions that in some languages the concept may be difficult to translate and that, after consideration in the group cognitive interview, a decision may be made to leave it out so as to avoid complicating the translation or making it less than clear. Another English phrase that was found to be systematically difficult to translate was *up-to-date* (Scale 8 – Arabic, both French translations, Hindi and Indonesian). However, rather than coding this as Idiom/literal meaning, it was coded as Complex word or phrase because the lead translators described that the changes were to make the translated words clearer for all and easier to understand.

## Discussion

Just as rigorous post-translation quantitative analysis is needed to determine if a PROM measures the same construct in the same way in two language versions [12–17], so too is rigour required during the translation process to qualitatively maximise the construct and measurement equivalence of items (and thus scales) between the languages. This study examined the routine documentation of 9 HLQ translations using the TIP and found that 259 errors had been made. These errors could have resulted in items that did not measure the same health literacy construct as the English HLQ. Threats to construct equivalence can lead to interpretations of data that are not valid and, subsequently, to potentially invalid and flawed decision making [3, 9, 10, 20, 26, 78]. Results from this study reinforce the need for a multi-step translation and central review process [3, 7, 20, 26, 32, 50]. In addition, this study has demonstrated that reference to documented item intent descriptions can support translation teams to detect even mildly nuanced errors in meaning between source and translated items. In fact, this study instigated a change in the TIP from two forward translators to one independent forward translator and one translator independently checking the forward translation against the item intents, then both translators conferring about differences. The use of item intents guided the translators about the meaning of words and terms so both linguistic and cultural aspects of the translation could be considered. Particularly evident was the detection of the high number of complex words and phrases used by translators, which may have prevented some respondents with low literacy levels from answering items in the same way as respondents with higher literacy levels. Also, words with similar but different meanings (e.g., find and get) that go undetected may create preventable challenges for construct equivalence. The group cognitive interviews provided the most in-depth discussions when local people (e.g., health workers or patients/consumers) worked with the translators. Local speakers of a language can detect nuanced and fine distinctions in meanings of words that professionally-trained translators can be unfamiliar with, and translators used to translating corporate or academic documents can use high level language that might make translated items inaccessible to target respondents [25]. Interestingly, there were very few cultural errors detected (*n* = 7), which perhaps indicates that the 9 HLQ scale constructs are relatively culturally neutral and the concepts transfer to these other languages and cultures with minimal construct underrepresentation bias to affect score interpretation.

Guidelines for linguistic and cultural adaptations of construct-based PROMs usually consist of a common set of components: forward translation to the target language by one or two translators; back translation to the source language by an independent translator; expert committee consultation; and cognitive interviews with members of the target population prior to consensus on the final translation and quantitative testing [1, 3, 21, 28, 32, 77, 79–82]. However, despite recommendations for translation methods to be reported as validity evidence [10, 53], there has been little formal research about how the components of translation methods contribute to construct equivalence between PROMs, and only rare but brief mention of how item intent descriptions might support translations to maximise construct equivalence [19, 21]. In a recent publication, Acquadro et al. include in the definition of translatability assessment (i.e., the important step of reviewing the suitability of a PROM for translation) that ‘alternative choices of wordings on which translations can be based’ should be provided [19]. The International Society for Pharmacoeconomics and Outcomes Research (ISPOR) Principles of Good Practice for the Translation and Cultural Adaptation of Patient-Reported Outcomes (PRO) Measures report makes brief mention that an ‘explanation of concepts’ in an instrument should be developed as part of the preparatory work for PROM translation [21]. The reason given for the need for the explanations of concepts is ‘to strengthen the conceptual equivalence of the forward translations, and help to avoid any ambiguities’ (Table 1. p.98) [21]. However, a greater emphasis is needed on the importance of such an explanatory document for the integrity of the translation process, especially if the PROM developer is unable to be part of the translation team. Item intent descriptions inform forward translators about what an item means (and sometimes what it does not mean) and forge a common ground from which translation team members can qualitatively strive to maximise construct equivalence prior to quantitative confirmation testing.

### Limitations to this study

Limitations to this study were that documentation of changes to the translated HLQs was reliant on detail provided by the translators, some of whom were more dedicated to the task of providing reasons for changes than others. Coding could only be applied according to the explanations offered by the translators, which was difficult or impossible if the explanation was scant or difficult to understand. This might mean that the TIP could detect more than 259 errors but these were not described such that they could be coded. Another limitation was not being able to report on the qualifications of the translators because the documentation did not require translators to provide their technical qualifications. However, as is seen by the most frequently coded error (Complex word or phrase), translation qualifications are not the most important aspect of the process for translations of PROMs for use with people with potentially low education and literacy levels. More important is a translator’s understanding of the language used by the everyday people in a target population or the attendance at the group cognitive interview of local people such as healthcare workers, patients or other consumers of healthcare services.

### Strengths of this study

A strength of this study was that the translators analysed the translated HLQ items according to the item intent descriptions and translators recorded the process of decision making during the group cognitive interviews, which enables a transparent translation method. Another strength was that the 9 languages covered a range of language groups (European, Asian and African), which indicates that this translation method can be applied in different linguistic and cultural contexts. The examination of the field data was a rigorous process that others can replicate to test other translation methods. Also, and importantly, a well-founded theoretical validity testing framework underpins the study rationale [10].

## Conclusions

To advance PROM translation practice, this study investigated the use of the Translation Integrity Procedure (TIP), a documented translation method that includes the careful specification of descriptions of item intents. Comparisons of cross-cultural PROM data rely on measurement invariance to produce unbiased estimates of mean differences across settings. Assumptions that translated PROMs have construct equivalence between linguistic contexts can be incorrect due to errors in translation. Evidence for the plausible justification of score interpretation and use of translated PROMS includes transparent documentation of the translation method [10, 23, 83]. The TIP and, in particular, item intent descriptions enable systematic translation documentation and a common foundation for translation teams to negotiate the nuances of item meanings so as to maximise construct equivalence, minimise threats to construct validity during the translation process, and generate qualitative validity evidence for score interpretation and use in a new linguistic context.

## Supplementary information

**Additional file 1.** ‘Translation Integrity Procedure (TIP) Version 5.pdf’ provides information about the translation procedure used in this study

## Data Availability

The datasets used and/or analysed during the current study are available from the corresponding author on reasonable request.

## Abbreviations

PROM: Patient-Reported Outcomes Measure
PRO: Patient-Reported Outcomes
The *Standards*: The *Standards for Educational and Psychological Testing*
TIP: Translation Integrity Procedure
HLQ: Health Literacy Questionnaire
DUHREC: Deakin University Human Research Ethics Committee
ISPOR: International Society for Pharmacoeconomics and Outcomes Research

## Footnotes

1 We use ‘patient-reported outcome measure’ (PROM) to encompass all health-related assessments that are based on individuals’ responses to self-report questionnaires, whether written or verbally administered.
