# Supplementary material for: Translation method is validity evidence for construct equivalence: analysis of secondary data routinely collected during translations of the Health Literacy Questionnaire (HLQ)
Source: BMC Med Res Methodol. 2020 May 26;20:130. doi: 10.1186/s12874-020-00962-8 (PMC7249296; doi:10.1186/s12874-020-00962-8)
Supplement: Supplementary file 1 — Additional file 1. ‘Translation Integrity Procedure (TIP) Version 5.pdf’ provides information about the translation procedure used in this study [file 12874_2020_962_MOESM1_ESM.pdf]

Translation Integrity Procedure (TIP)

For the translation and cultural adaptation of psychometric questionnaires

Version 5

(May 2017)

Authors:

Melanie Hawkins

Richard H Osborne

Email: Richard Osborne - rosborne@swin.edu.au

Contents

1. INTRODUCTION TO THE TRANSLATION INTEGRITY PROCEDURE (TIP) 3

1.1 TRANSLATION LICENCE 3
1.2 BACKGROUND – CONSTRUCT EQUIVALENCE BETWEEN SOURCE AND TARGET LANGUAGES 3
1.3 THE TRANSLATION TEAM 4
The core team 4
Other members of the translation team 5
1.4 THE GROUP COGNITIVE INTERVIEW 5
Figure 1. Translation Integrity Procedure flow diagram 6

2. THE TRANSLATION INTEGRITY PROCEDURE (TIP) 7

2.1 FORWARD TRANSLATION 7
2.2 BACK TRANSLATION 8
2.3 PREPARATION FOR THE GROUP COGNITIVE INTERVIEW 8
2.4 THE GROUP COGNITIVE INTERVIEW 9
Figure 2. Group cognitive interview – participants 10

3. VALIDITY TESTING OF THE TRANSLATED QUESTIONNAIRE 11

4. SUGGESTED READING 12

5. REFERENCES 14

1. Introduction to the Translation Integrity Procedure (TIP)

This manual describes the Translation Integrity Procedure (TIP). The TIP guides researchers, program managers and translators to produce translations of psychometric questionnaires that are conceptually equivalent to the source language questionnaire, are appropriate for the target country and/or cultural group, use natural and acceptable language and phrasing, are easily read and understood by people with low literacy, and demonstrate a measurement performance that is equivalent to the source questionnaire. The TIP has been tested across many languages and over time (see Section 4). The procedure is outlined in detail in Section 2.

1.1 Translation licence

Please note that a translation licence may be required for the questionnaire you wish to translate. Always contact the authors of any questionnaire you plan to translate prior to the translation to determine copyright and intellectual property issues. Fees may be involved.

1.2 Background – construct equivalence between source and target languages

A core difference between the translation of technical documents (such as letters and reports) and psychometric questionnaires is the need to maximise measurement equivalence between the source and the target language questionnaires, as well as to accommodate linguistic and cultural adaptation. This section explains the importance of having a systematic and documented translation method to qualitatively maximise construct equivalence, and to contribute evidence to justify the score interpretations for use in the new linguistic context.

Psychometric questionnaires consist of one or more constructs (i.e., abstract concepts), each of which is represented by a psychometric scale. Each scale consists of items (questions or statements that require a response) that measure elements of its construct. Items work together within a scale to measure specific and independent constructs. Respondents usually score the items along an ordinal scale (e.g., response options that range from ‘strongly agree’ to ‘strongly disagree’).

To achieve accurate measurement across the full continuum of a person’s status, a questionnaire scale will typically have 4 to 6 items that measure varying strengths of the elements of the construct. That is, scale items are designed to make it easier or more difficult for respondents to positively endorse those items. So translators must be mindful of maintaining the same item meanings and maintaining the same range of measurement relationships between items in a scale.

It is a considerable challenge to achieve equivalence of abstract constructs across different languages and cultures, especially when languages can be linguistically and conceptually very different. It is the task of a translation team to ensure that the translation method

maximises the measurement equivalence of items (and thus of constructs) between the source and target languages. Intended users of a questionnaire must be assured that the items of the translated questionnaire will measure the same constructs in the same way as the source language questionnaire when used for the same purpose and under the same circumstances (same context). An analogy for capturing the same construct in two languages is that of measuring characteristics of water in Celsius and in Fahrenheit. A thermometer in Celsius has a scale that measures frozen water at 0°C (an extreme endpoint) through gradations of temperatures to boiling at 100°C (the other extreme endpoint). A score on a Fahrenheit scale for frozen (32°F) and a score for boiling (212°F) still measure the same characteristics of water even though the 'language' to name the condition is different.

An example to help understand the relationships between items within a scale is when measuring a condition such as depression: the items would be designed to detect extreme depression (that is, suicidal, long-term intractable negative mood, profound adverse effect on the person's life) through to moderate and then to very mild depression (that is, occasional and transient feelings of sadness, but mostly positive mood). For each scale, there needs to be items that detect small differences in the strength of a characteristic along any part of the scale, as well as improvements or decrements over time. The translation process must maintain not only the meaning of the source items but also the varying strengths of the characteristics of the constructs (i.e., the measurement relationships between items).

A translated questionnaire will require cognitive testing in the target language, and testing for psychometric comparison with the source language questionnaire prior to use in measurement studies in the target language (see Sections 3 and 4).

1.3 The translation team

The core team

The translators need high-level technical qualifications and/or comprehensive experience with written translation of the target language and, preferably, knowledge of the nuances of local dialects. Translations need to achieve the breadth and depth of local language variations and pursue the use of simple text that can be easily understood by all people, including those with only modest language skills. This is achieved through the support of bilingual lay people, healthcare workers from the field, and public health professionals on the translation team. It is important to ensure that the original constructs of a questionnaire are properly represented by the items in the target language through linguistic, cultural and measurement equivalence.

Two forward translators and one back translator are required. **The forward translators are native speakers of the target language**, have verified technical qualifications for translation and are fluent in the source language. One forward translator uses the item intents to translate

the questionnaire to the target language. The second translator checks the translation of each item against the item intents. Any discrepancies in translation are discussed, using the item intents as the guide, until agreement of the provisional forward translation is reached.

The back translator is a native speaker of the source language with excellent knowledge of and fluency in the target language. It is essential that the back translator does not see the source items so that a blind back translation can be undertaken. The back translation is used by anyone in the translation team who does not know the target language. This back translation will be used by the chairperson to gain an understanding of discrepancies or errors that may be occurring in the forward translation.

Other members of the translation team

It is advantageous to have several **bilingual health workers and/or laypeople with knowledge of and an interest in local language and culture**. Some countries have several dialects of a language and many local cultural beliefs. The quality of the translation is optimised when bilingual health workers and/or lay people participate in discussions about the most commonly understood wording and phrasing of questions, and about local customs that may affect the translation. The presence of **a native source language speaker who is fluent in the target language** enables clarification by the chairperson of source language nuances of translated words. This person may be one of the translators.

1.4 The group cognitive interview

The goal of the group cognitive interview is for the translation team to examine translated items against corresponding item intent descriptions, which are included in the Item Intent and Translation Management Grid (the Grid). Discrepancies in meaning between the translated items and the source items are negotiated to maximise equivalence in meaning and measurement. That is, the items in the two language versions must, as closely as possible, measure the same constructs in the same way. There must be construct equivalence to achieve equivalent interpretations of data from a translated questionnaire, and for the data to be comparable across language and cultural settings. Analysis of documented data from a TIP translation method contributes evidence to an evaluation of the extent to which score interpretations are valid for the new linguistic context.

Required at the group cognitive interview are the lead forward translator and one other translator, the native speaker of the source language (fluent in the target language), and the chairperson who will lead the group cognitive interview. Other attendees can include bilingual field workers and/or lay people with an interest in the local language and culture.

See next page for the TIP flow diagram (Figure 1).

Figure 1. Translation Integrity Procedure flow diagram

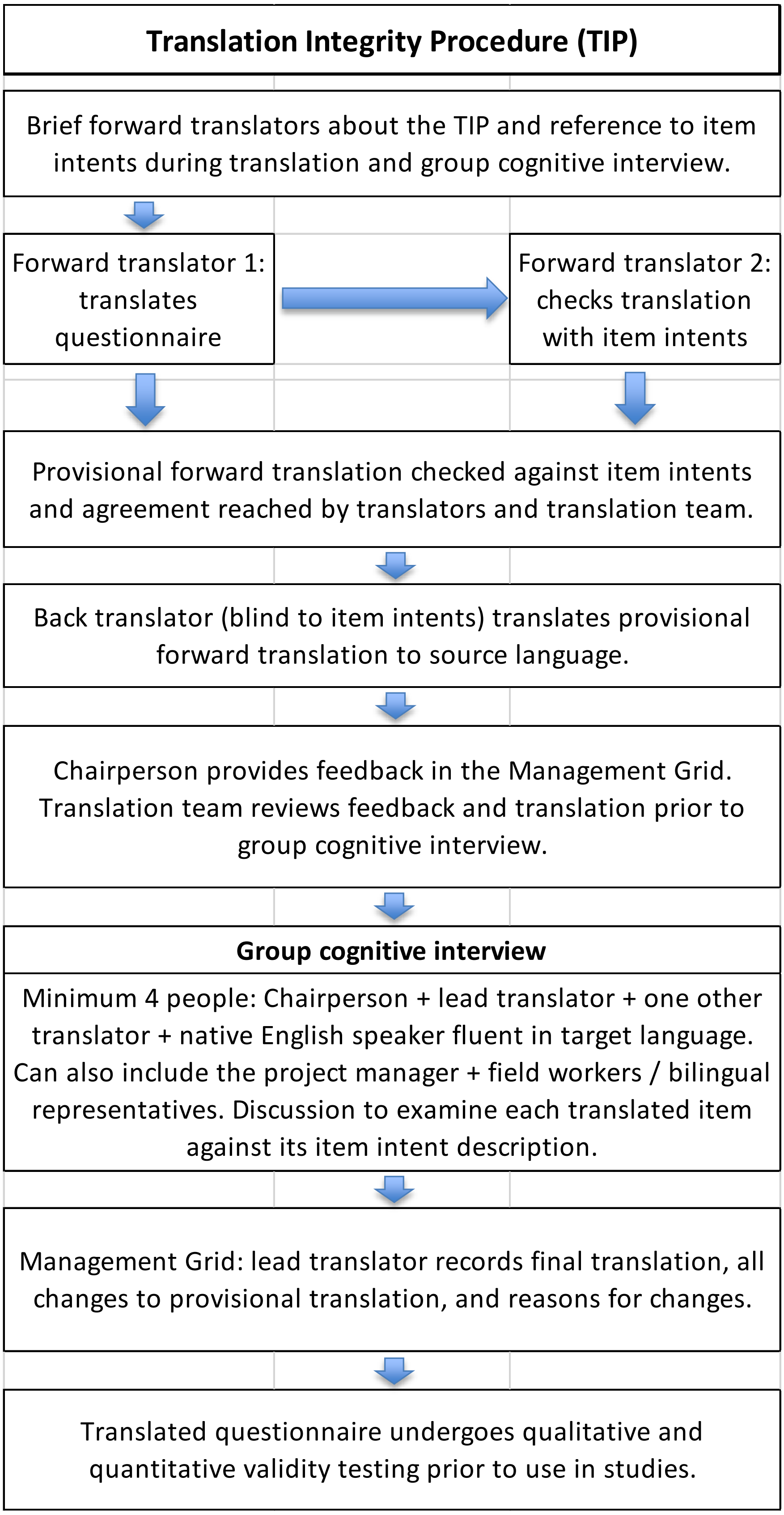

2. The Translation Integrity Procedure (TIP)

A TIP translation package includes the following documents:

a) A memo to the translators from the chairperson emphasising the use of item intents
b) The Item Intent and Translation Management Grid
c) A PDF formatted version of the questionnaire in the source language
d) A Word formatted version of the questionnaire in the source language, which is the template into which the final agreed translation is formatted (note that the introduction and instructions on the template will also require translation)

The memo to translators is a welcome note and it outlines the expectations for the translation. The forward translators are to be briefed about the development and purpose of the questionnaire, and given opportunity to clarify questions they may have as to the intent or meaning of items. Good communication in the translation team enables translators to better understand the client's expectations and to provide a high quality translation.

The Grid is the main working document for the translation process and provides a format within which to manage the forward and back translation. It is the primary working document for the group cognitive interview. It contains descriptions of high and low scoring for each of the constructs, and of the intent of each item in the questionnaire. The items are grouped within their constructs. Forward translators use the item intent descriptions to guide their translation and to pursue, as closely as possible, the meaning and measurement relationships of the source items. The goal of the item intent descriptions is to support translators to gain an in-depth understanding of the meaning conveyed in each item and gives alternative words to consider if the concept does not translate exactly to the target language.

Please note that the back translator must remain blind to the items and item intents, so the forward translation must not be sent in the Grid to the back translator.

2.1 Forward translation

There are two forward translators. Both are native speakers of the target language with very high levels of proficiency (fluency) in the source language. The forward translators must continually refer to the item intent descriptions during the translation. The lead translator first translates the items from the source language to the target language. This translation is then checked by the second forward translator. Both translators then confer, with reference to the item intent descriptions, to decide on an agreed provisional forward translation. They need to agree that the translation is as close to the conceptual meaning of the source questionnaire as can be accomplished, that I can be understood by people with low literacy skills, and that it uses natural language that is appropriate to the country or culture of the

target language group. Furthermore, the number of words must be kept to a minimum to reduce the burden on people completing the questionnaire.

If there is persistent discrepancy between the forward translators about how an item or concept should be translated then they can offer alternatives for the back translation. In some circumstances, the alternative items can be later pilot tested with respondents to establish the wording that best conveys the meaning of the source item. When both the forward translators are satisfied with the translated document, the provisional forward translation can then be passed to the back translator. It is very important that the back translator is kept blind to the source items and the item intent descriptions.

2.2 Back translation

The back translator is a native speaker of the source language with high proficiency (fluency) in the target language. **The back translator must be kept blind to the source items** while translating the provisional forward translation back into the source language. The main purpose of the back translation is for use in the group cognitive interview when the chairperson and/or other team members do not speak the target language. The back translation will help alert the chairperson to discrepancies in meaning between the source language items and the provisional forward translation. These discrepancies can then be discussed with the forward translators to arrive at a final consensus translation.

2.3 Preparation for the group cognitive interview

Prior to the group cognitive interview, the study manager prepares the Grid with both forward and back translations and sends it to the chairperson. The chairperson provides feedback on the back translation and inserts this into the Grid. The chairperson will examine the back translation for all aspects of each translated item, especially the relative strength of the wording between items and how the items work together within the scale. The chairperson may challenge the choice of words used by translators. The chairperson must understand the translation process and be alert to the issues that can arise. For example:

- Linguistic issues – the lexical, syntactic or semantic demands of the target language appear to cause discrepancies between the meaning of a source language item and the translated item
- Back translation issues – the back translation is incorrect or has not offered a suitable alternative, and causes concern about the meaning conveyed in the translated item
- Translation issues – the back translation accurately identifies errors in the provisional forward translation that need to be corrected

The Grid with the chairperson's feedback is returned to the client to be distributed to all

translators. Each translator reviews the feedback separately and records their comments in the Grid. This is an opportunity for the translators to develop explanations for, or corrections to, their translations, including agreement or disagreement with the other translators and the chairperson. All areas for concern are clearly highlighted and made ready for the translation team to discuss during the group cognitive interview.

During the meeting, it is important to not dwell on the back translation. It is only used to support negotiation of the forward translation. It will be disregarded after the meeting.

2.4 The group cognitive interview

The aim of this meeting is for the chairperson to determine the quality of the translation. This occurs through in-depth discussions with the translation team about the meanings of translated words or phrases to determine if the translated items convey the same or comparable meanings as the source language items. Depending on the quality of the translation (i.e., adherence to the item intents), the discussions will take between 3 and 5 hours. The final translation is arrived at through these negotiations between the chairperson and the translation team. The translated items are then ready to undergo validity testing.

The following table outlines the participants who need to attend the group cognitive interview.

Figure 2. Group cognitive interview – participants

This form is included in the Item Intent and Translation Management Grid document.

** Mandatory participants
* Highly recommended participants

Participant		Name	Role	Country	Time zone / Skype address	Local time
1	Chairperson **		*Chair of the meeting*			
2	Forward translator 1**		*Primary spokesperson and* ***responsible for recording agreed changes***			
3	Forward translator 2**		*Secondary spokesperson*			
4	Native speaker of source language and fluent in target language**		*Spokesperson*			
5	Study management*		*Discussant*			
6	Local bilingual or local language representatives*		*Discussants*			
7	Others as needed*		*Discussants*			

3. Validity testing of the translated questionnaire

Approaches to questionnaire development and validity testing are described elsewhere [1-5]. Validity evidence for a translated questionnaire can include data from analysis of the documented translation process, cognitive interviews with the target population, and psychometric analyses to compare data with validity studies of the source language questionnaire. Evaluation of these types of validity evidence will determine the extent to which the intended interpretation of scores is valid for the intended use in the new linguistic context.

It is recommended that existing or newly generated evidence from five sources be considered for the evaluation of the validity of score interpretation and use in a new context [5]:

1. Evidence based on test content – the relationship of the item themes, wording and format with the intended construct, including administration process

2. Evidence based on response processes – the cognitive processes and interpretation of items by respondents and users, as measured against the intended construct

3. Evidence based on internal structure – the extent to which item interrelationships conform to the intended construct

4. Evidence based on external variables – the pattern of relationships of test scores to external variables as predicted by the intended construct

5. Evidence based on validity and the consequences of testing – intended and unintended consequences, as can be traced to a source of invalidity such as construct underrepresentation or construct-irrelevant variance

Analysis of data from a documented translation process such as the TIP contributes qualitative evidence for test content, response processes, and internal structure of a translated questionnaire. Translation method should help to maximise construct equivalence during the translation. Cognitive interviews that compare interview narratives with item intent descriptions determine if respondents are engaging with the intended construct of measurement [5-7]. Feedback from the target respondents can provide rich information about the appropriateness of the language used, the concepts conveyed, and the suitability for the target country or culture. Quantitative statistical validation is required to test the psychometric properties of a translated questionnaire against those of the source language version.

An evidence-based argument for the extent to which score interpretations of the translated questionnaire are valid for the intended use is needed before conducting measurement studies in the field.

4. Suggested reading

heiQ

1. Osborne RH, Elsworth GR, Whitfield K. The Health Education Impact Questionnaire (heiQ): An outcomes and evaluation measure for patient education and self-management interventions for people with chronic conditions. *Patient Education and Counseling* 2007; 66(2): 192-201 http://www.sciencedirect.com/science/article/pii/S0738399106004216
2. Elsworth GR, Nolte S, Osborne RH. Factor structure and measurement invariance of the Health Education Impact Questionnaire: Does the subjectivity of the response perspective threaten the contextual validity of inferences? *Sage Open Medicine* 2015; 3. https://www.ncbi.nlm.nih.gov/pmc/articles/PMC4679238/
3. Nolte S, Elsworth GR, Osborne RH. Absence of social desirability bias in the evaluation of chronic disease self-management interventions. *Health and Quality of Life Outcomes* 2017; 11:114 http://hqlo.biomedcentral.com/articles/10.1186/1477-7525-11-114

HLQ

4. Osborne RH, Batterham R, Elsworth G, Hawkins M, Buchbinder R. The grounded psychometric development and initial validation of the Health Literacy Questionnaire (HLQ). *BMC Public Health* 2013; 13:658. http://www.biomedcentral.com/1471-2458/13/658
5. Hawkins, M., G.R. Elsworth, and R.H. Osborne, Application of validity theory and methodology to patient-reported outcome measures (PROMs): building an argument for validity. Quality of Life Research, 2018: p. 1-16. https://doi.org/10.1007/s11136-018-1815-6
6. Bo A, Friis K, Osborne RH, Maindal HT. National indicators of health literacy: ability to understand health information and to engage actively with health care providers – a population-based survey among Danish adults. *BMC Public Health* 2014; 14:1095 doi: 10.1186/1471-2458-14-1095 http://bmcpublichealth.biomedcentral.com/articles/10.1186/1471-2458-14-1095
7. Beauchamp A, Buchbinder R, Dodson, Batterham RW, Elsworth GR, McPhee C, Sparkes L, Hawkins M, Osborne RH. Distribution of health literacy strengths and weaknesses across socio-demographic groups: a cross-sectional survey using the Health Literacy Questionnaire (HLQ). BMC Public Health 2015; 15:678 http://www.biomedcentral.com/1471-2458/15/678
8. Batterham RW, Hawkins M, Collins PA, Buchbinder R, Osborne RH. Health literacy: applying current concepts to improve health services and reduce health inequalities.

Public Health 2016; 132: 3-12 http://www.sciencedirect.com/science/article/pii/S0033350616000044

9. Friis K, Lasgaard M, Rowlands J, Osborne RH, Maindal HT. Health Literacy Mediates the Relationship Between Educational Attainment and Health Behavior: A Danish Population-based Study. Journal of Health Communication 2016; DOI: 10.1080/10810730.2016.1201175 http://www.tandfonline.com/doi/abs/10.1080/10810730.2016.1201175
10. Maindal HT, Kayser L, Norgaard O, Bo A, Elsworth GR, Osborne RH. Cultural adaptation and validation of the Health Literacy Questionnaire (HLQ): robust nine-dimension Danish language confirmatory factor model. SpringerPlus 2016; 5:1232 doi: 10.1186/s40064-016-2887-9 https://springerplus.springeropen.com/articles/10.1186/s40064-016-2887-9
11. Elsworth GR, Beauchamp A, Osborne RH. Measuring Health Literacy in Community Agencies: A Bayesian Study of the Factor Structure and Measurement Invariance of the Health Literacy Questionnaire (HLQ). BMC Health Services Research 2016; 6(1):508 DOI 10.1186/s12913-016-1754-2 https://bmchealthservres.biomedcentral.com/articles/10.1186/s12913-016-1754-2?utm_campaign=BMC_TrendMD&utm_medium=cpc&utm_source=TrendMD
12. Kolarčik P, Elsworth G, Batterham R, Osborne RH. Structural properties and psychometric improvements of the Health Literacy Questionnaire (HLQ) in a Slovak population. *International Journal of Public Health* 2017; DOI 10.1007/s00038-017-0945-x
13. Jessup R, Osborne RH, Beauchamp A, Buchbinder R. Health literacy of recently hospitalised patients: a cross-sectional survey using the Health Literacy Questionnaire (HLQ). *BMC Health Services Research* 2017; 17:52 DOI: 10.1186/s12913-016-1973-6 https://bmchealthservres.biomedcentral.com/articles/10.1186/s12913-016-1973-6
14. Nolte S, Osborne RH, Dwinger S, Elsworth GR, Conrad ML, Rose M, Härter M, Dirmaier J, Zill JM. German translation, cultural adaptation, and validation of the Health Literacy Questionnaire (HLQ). 2017; *PLOS ONE* 12(2): e0172340. doi: 10.1371/journal.pone.0172340 http://journals.plos.org/plosone/article?id=10.1371/journal.pone.0172340
15. Hawkins M, Gill S, Batterham R, Elsworth GR, Osborne RH. The Health Literacy Questionnaire (HLQ) at the patient-clinician interface: a qualitative study of what patients and clinicians mean by their HLQ scores. BMC Health Services Research 2017; 17:309 https://bmchealthservres.biomedcentral.com/articles/10.1186/s12913-017-2254-8

5. References

1. Osborne, R.H., G.R. Elsworth, and K. Whitfield, *The Health Education Impact Questionnaire (heiQ): an outcomes and evaluation measure for patient education and self-management interventions for people with chronic conditions.* Patient Education and Counseling, 2007. **66**(2): p. 192-201.
2. Buchbinder, R., et al., *A validity-driven approach to the understanding of the personal and societal burden of low back pain: development of a conceptual and measurement model.* Arthritis Research & Therapy, 2011. **13**(5): p. R152.
3. Osborne, R.H., et al., *The grounded psychometric development and initial validation of the Health Literacy Questionnaire (HLQ).* BMC Public Health, 2013. **13**: p. 658.
4. Hawkins, M., G.R. Elsworth, and R.H. Osborne, *Application of validity theory and methodology to patient-reported outcome measures (PROMs): building an argument for validity.* Quality of Life Research, 2018: p. 1-16.
5. American Educational Research Association, American Psychological Association, and National Council on Measurement in Education, *Standards for educational and psychological testing*. 2014, Washington, DC: American Educational Research Association.
6. Kane, M.T., *An argument-based approach to validity.* Psychological Bulletin, 1992. **112**(3): p. 527-535.
7. Kane, M.T., *Validating the interpretations and uses of test scores.* Journal of Educational Measurement, 2013. **50**(1): p. 1-73.
